# Quantitative screening of the effects of hyper-osmotic stress on cancer cells cultured in 2- or 3-dimensional settings

**DOI:** 10.1038/s41598-019-50198-w

**Published:** 2019-09-24

**Authors:** Agnes Miermont, Sharon Wei Ling Lee, Giulia Adriani, Roger D. Kamm

**Affiliations:** 1 Stem Genomics, IRMB, Univ Montpellier, INSERM, CHU Montpellier, Montpellier, France; 2 BioSystems and Micromechanics, IRG, Singapore-MIT Alliance for Research and Technology, Singapore, Singapore; 3 Department of Microbiology and Immunology, Yong Loo Lin School of Medicine, National University of Singapore, Singapore, Singapore; 4 Singapore Immunology Network (SIgN), Biomedical Sciences Institute, Agency for Science, Technology and Research (A*STAR), Singapore, Singapore; 5 Department of Biomedical Engineering, Faculty of Engineering, National University of Singapore, Singapore, Singapore; 6 Department of Biological Engineering, Massachusetts Institute of Technology, Cambridge, MA United States

**Keywords:** Cell invasion, Cancer microenvironment

## Abstract

The maintenance of precise cell volume is critical for cell survival. Changes in extracellular osmolarity affect cell volume and may impact various cellular processes such as mitosis, mitochondrial functions, DNA repair as well as cell migration and proliferation. Much of what we know about the mechanisms of cell osmoregulation comes from *in vitro* two-dimensional (2D) assays that are less physiologically relevant than three-dimensional (3D) *in vitro* or *in vivo* settings. Here, we developed a microfluidic model to study the impact of hyper-osmotic stress on the migration, proliferation and ion channel/transporter expression changes of three metastatic cell lines (MDA-MB-231, A549, T24) in 2D versus 3D environments. We observed a global decrease in cell migration and proliferation upon hyper-osmotic stress treatment, with similar responses between 2D and 3D conditions. Specific ion channels/aquaporins are over-expressed in metastatic cells and play a central role during osmo-regulation. Therefore, the effects of hyper-osmotic stress on two transporters, aquaporin 5 (AQP5) and the transient receptor potential cation channel (TRPV4), was investigated. While hyper-osmotic stress had no major impact on the transporters of cells cultured in 2D, cells embedded in collagen gel (3D) decreased their AQP5 expression and exhibited a reduction in intra-cellular translocation of TRPV4. Furthermore, cell dispersion from T24 aggregates embedded in 3D collagen gel decreased with higher levels of hyper-osmotic stress. In conclusion, this study provides evidence on the impact of hyper-osmotic stress on various aspects of metastatic cell progression and highlights the importance of having a 3D cell culture platform in investigating molecular players involved in cancer cell migration.

## Introduction

The first step of the metastatic cascade is the dissociation of cancer cells from the tumor and their migration through the stroma toward the blood stream^1,2^. Migration is, therefore, a critical step for metastatic progression. The classical model for cell migration, involving protrusions driven by actomyosin polymerization and contraction, arises from *in vitro* studies conducted on rigid two-dimensional (2D) surfaces^3–5^. However *in vitro* models that involve the culture of cells as monolayers on a flat surface fail to recapitulate the physiological environment of a metastatic cell migrating in a three-dimensional (3D) extracellular matrix (ECM). Therefore, there is need for improved *in vitro* 3D models that investigate cell migration.

In the past decade, different mechanisms of cancer cell migration have been extensively described and are known to depend on gene expression profiles and signalling cues as well as mechanical properties of the matrix^6–8^. Also, the mode of migration of cancer cells is influenced by the modification of the ECM tension and stiffness as well as the presence of small pores^9,10^. Based on this, Pathak *et al*. showed that the migration speed of a confined cell increases with the stiffness of the environment^11^. Other parameters such as mechanical compression can critically impact cancer cell migration and proliferation, and their growth in the form of tumor spheroids^12–14^. Moreover, extracellular osmolarity or modification of specific ion concentrations strongly impacts the mechanical properties of the cell through cell volume changes that may affect various cellular processes (e.g. mitosis, mitochondrial functions and DNA repair)^15–19^.

Previous studies showed that cancer cells are sensitive to hyper-osmotic stresses which resulted in a decrease in cell motility, transmigration capacities and proliferation^12,20^. However, metastatic cancer cells have been described to be less sensitive to hyper-osmotic stresses than primary tumor cancer cells^12^. One possible reason for the differences observed is the implication of specific ion pumps and aquaporins. Indeed, these transporters - which play crucial roles in osmoregulation – are associated with metastatic status^21,22^ and are involved in cancer cell migration through confined spaces^23^.

*In vivo*, cells are surrounded by the extracellular matrix which is composed of fibrillar collagen forming pores of various sizes that affect signal transduction, gene regulation, cellular behavior as well as cell morphology. Those modifications are necessary for the cells to adapt to different topology and rigidity to successfully transmigrate. Because 2D models lack the physical constraints and spatial organization of the cell in its microenvironment, traditional monolayer cultures are less physiologically relevant than 3D matrix to study biological mechanisms such as tumor cell migration or response to various stimuli^24,25^.

However, although previous studies provided essential clues on osmoregulation and cancer cell migration, the precise impact of osmotic stress on metastatic cells cultured in 3D is still poorly understood. This is related to the paucity of appropriately designed models to study the effect of cell migration under the exposure to different mechanical stimuli. For example, Stroka *et al*. used a confined 3D environment consisting of fixed diameter microfluidic channels which looked solely at the effect of transient osmotic stresses applied on each side of the cell^23^. On the other hand, La Porta *et al*. used classical transwell assays which do not capture the physiological complexity of a 3D setting and do not allow for dynamic high resolution single-cell imaging^12^.

**Figure 1:**
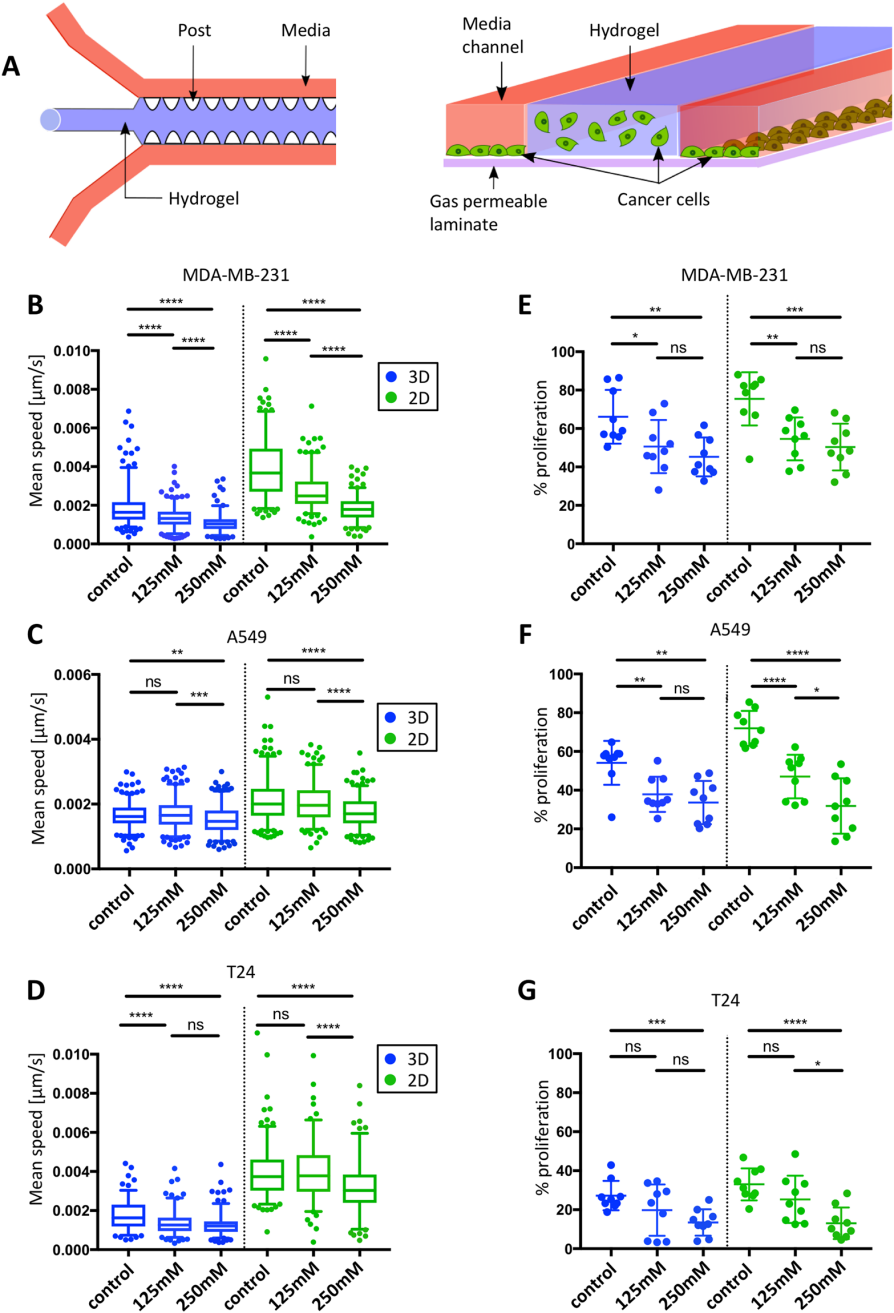
Impact of hyper-osmotic stress on cancer cell migration speed and proliferation in 3D and 2D conditions. (**A**) Schematic illustration (left image) of a 3D cell culture chip consisting of a central hydrogel region (blue) flanked by two media channels (orange) separated by PDMS posts (white). On the right is a cross-section of the microfluidic device. Cancer cells (in green) were simultaneously embedded in the middle channel (3D) and seeded in the two lateral fluidic channels (2D), allowing direct comparison of cell migration and proliferation behavior in both 3D and 2D conditions. (**B**–**D**) Average cell migration speed of MDA-MB-231 cells (**B**), A549 cells (**C**) and T24 cells (**D**) subjected to different hyper-osmotic conditions in either 3D (blue plots) or 2D (green plots) environments. The median (whisker boxes) and 5^th^ to 95^th^ percentile (lines) are shown for each condition. (**E**–**G**) Proliferation rate of MDA-MB-231 cells (**E**), A549 cells (**F**) and T24 cells (**G**) subjected to different hyper-osmotic conditions in either 3D (blue plots) or 2D (green plots) environments. Legend box is shown once for each cell line (**B**–**D**). Data are presented as the mean ± SD.

Therefore, in this project, an *in vitro* 3D microfluidic device has been used to mimic the physical and spatial characteristics of a tumor microenvironment. The main advantage of this model is the possibility to simultaneously perform real-time imaging of cells cultured in 3D or 2D settings by embedding cancer cells in the central hydrogel region while seeding the same cell type without hydrogel in the lateral fluidic channels^26–28^ (Fig. 1A). This system allows for the characterization of the impact of environmental changes – in our case hyper-osmotic stress - on the migration and proliferation capacities of cancer cells cultured in the two different conditions.

This study further investigates the expression of aquaporin 5 (AQP5) and the transient receptor potential cation channel (TRPV4) as these are highly involved in osmo-regulation. Water channel aquaporins are transmembrane proteins which play an essential role in cellular water transport and volume regulation. The over-expression of various AQPs has been observed in many tumor tissues^29,30^. Among those, AQP5 expression has been associated with increased proliferation in liver cancer^31^ and with increased invasion in various cancer cell types^32^. Potential Vanilloid subtype 4 (TRPV4) is a calcium permeable channel which is activated by various physical and chemical stimuli including cell swelling^33^. Recent studies described the role of TRPV4 in tumor angiogenesis^34^ and its contribution to the migration and extravasation of breast cancer cells^21^.

Specifically, we described how changes in AQP5 expression and TRPV4 intra-cellular localisation under hyper-osmotic stress depend on the culture condition of different type of cancer cells. Finally, by embedding human urinary bladder carcinoma cell (T24) aggregates in 3D collagen structure, the impact of hyper-osmotic stress on multicellular structures was investigated. We observed that subjecting T24 aggregates to a uniform osmotic compression for 24 h only affected their dispersion but not their proliferation.

In summary, this study provides evidence on the impact of hyper-osmotic compression on the cellular responses of metastatic cells, both at the single cell level and when cells are in the form of 3D aggregates. In addition, the systematic comparison of cells cultured in 3D versus 2D permits an appreciation of the importance of a 3D setting when investigating cellular and molecular responses to external stresses.

## Results

### Impact of hyper-osmotic stress on cancer cell migration and proliferation

Three metastatic cell lines were compared, namely human breast adenocarcinoma cells (MDA-MB-231), human lung adenocarcinoma cells (A549) and human bladder carcinoma cells (T24). In order to simultaneously capture the response of cancer cells in a 3D versus 2D environment, mannitol was added into the side channels to induce either a moderate (125 mM mannitol; 469 mOsm/kg) or severe (250 mM mannitol; 608 mOsm/kg) hyper-osmotic stress level following values established in previous publications^35,36^. Cell response was recorded after 24 h of stress exposure and compared with a control condition (DMEM; 337 mOsm/kg). First, a significant decrease in cell speed was observed proportional to the level of stress applied in both 3D and 2D conditions for all the three cell lines tested (Fig. 1B–D). Then, MDA-MB-231 cells presented the highest sensitivity to hyper-osmotic stress in both 3D and 2D conditions (27.2 % and 42.2 % decrease for, respectively, 125 mM and 250 mM mannitol in 3D; and 30.1 % and 52.7 % decrease in 2D) (Table 1 and Fig. 1B). All three cell lines in 3D had significantly lower migration speed than cells in 2D when comparing identical conditions (*P* < 0.0001) (Fig. 1B–D).

**Table 1:** Summary of the percentage of mean speed change (compared to control condition) for the three metastatic cell lines.

% Mean speed change	3D		2D	
	125 mM	250 mM	125 mM	250 mM
MDA-MB-231	−27.181	−42.183	−30.106	−52.673
A549	2.587	−6.762	−1.307	−15.743
T24	−20.421	−26.888	−1.116	−17.032

Measuring cell proliferation, we observed a significant decrease in both 3D and 2D proportional to the level of stress applied for the three cell lines tested (Fig. 1E–G). MDA-MB-231 and T24 cells presented no significant difference between 2D and 3D situations for the tested conditions (MDA-MB-231 control *P* = 0.1737, 125 mM *P* = 0.5088, 250 mM *P* = 3483; T24 control *P* = 0.1383, 125 mM *P* = 0.3663, 250 mM *P* = 0.9); A549 cells showed only significant difference for the control condition (*P* = 0.0020), but no differences in hyper-osmotic conditions (125 mM *P* = 0.753, 250 mM *P* = 0.7718). For the three metastatic cell lines, proliferation was slightly more affected by hyper-osmotic stress when the cells were in a 2D compared to 3D environment.

We then questioned whether the cell sensitivity to hyper-osmotic stress in our system was related to a disruption in cell mechanical properties. Indeed, the first response to hyper-osmotic stress is a water efflux leading to cell shrinkage, which is known to cause cell stiffening^37^. The subsequent activation of various osmotically- or volume-activated ion channels is essential to restore a normal cell size. This response is rapid and cell volume is expected to recover its initial value few minutes to hours after moderate induction^38^. Therefore, we measured the volume of the different cell types 24 h after continuous hyper-osmotic induction and we observed no significant changes in cell volume in the 2D condition for all three metastatic cell lines (Fig. S1A–C). In the 3D condition, both MDA-MB-231 and A549 showed a small increase in cell volume from normal to moderate osmotic conditions but no changes from normal to severe osmotic stress was observed (Fig. S1A,B). Interestingly, T24 was the only cell line to show a decrease in cell volume after both moderate and severe osmotic stresses (Fig. S1C). Therefore, except for T24 cells in the 3D condition, the changes in cell speed and proliferation observed 24 h after hyper-osmotic induction did not seem to be directly related to cell volume changes.

### Hyper-osmotic stress induces a significant decrease in actin and AQP5 expression

Hyper-osmotic stress is known to impair cytoskeleton and actin rearrangement^39^. After 24 h of hyper-osmotic induction, we observed a decrease in actin expression in both 3D and 2D conditions (Fig. S1D–G). Cells embedded in 3D hydrogel showed higher actin intensities than in 2D for the same osmotic condition. We postulate that this variation, along with slower migration speed in 3D (Fig. 1B–D), arises from the difference in cell morphology and migration strategies adopted by cells in different culture conditions, with cells in 3D being smaller and showing less spread than the same cells on a flat isotropic 2D substrate^24^. Similar to the migration and proliferation results, different cell type sensitivity was observed, with MDA-MB-231 cells being the most responsive to osmotic variations.

**Figure 2:**
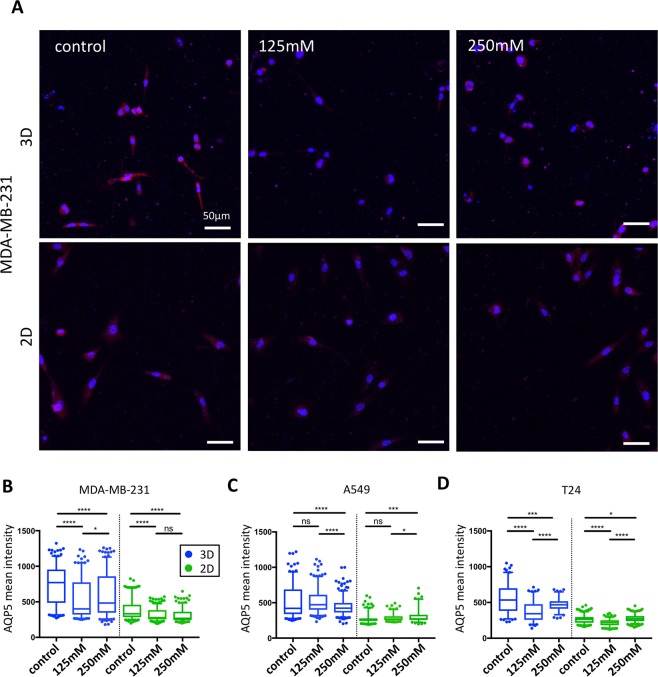
Changes in AQP5 expression under hyper-osmotic stress in 3D and 2D conditions. (**A**) Immunofluorescence staining of AQP5 in MDA-MB-231 cells under different hyper-osmotic conditions in 3D (top images) and 2D (bottom images). Cell nuclei are shown in blue and AQP5 in red. (**B**–**D**) Averaged AQP5 intensity per cell for MDA-MB-231 (**B**), A549 (**C**) and T24 cells (**D**) in either 3D (blue plots) or 2D (green plots) environments. The median (whisker boxes) and 5^th^ to 95^th^ percentile (lines) are shown for each condition. Legend box is shown once for all graphs.

Using immunofluorescence techniques, we then analysed how hyper-osmotic stress impacts AQP5 expression. We observed a significant decrease in AQP5 intensity in MDA-MB-231 cells in both 3D and 2D conditions (Fig. 2A,B). Despite being less affected than MDA-MB-231 cells, T24 and A549 also presented a significant decrease in AQP5 expression in the 3D condition (Figs 2C,D and S2). However, in 2D, the changes observed in A549 and T24 presented an opposite trend, with a slight increase in AQP5 intensity under severe hyper-osmotic stress compared to the control. Unlike previous migration and proliferation results, the AQP5 expression changes were not proportional to the stress applied, with moderate osmotic stress showing either an increased expression compared to control (A549 cells in 3D) or severe osmotic stress presenting an increased expression compared to moderate stress stimulus (T24 cells in both culture conditions, MDA-MB-231 cells in 3D).

### Change in intra-cellular localisation of TRPV4 under hyper-osmotic stress in 3D

**Figure 3:**
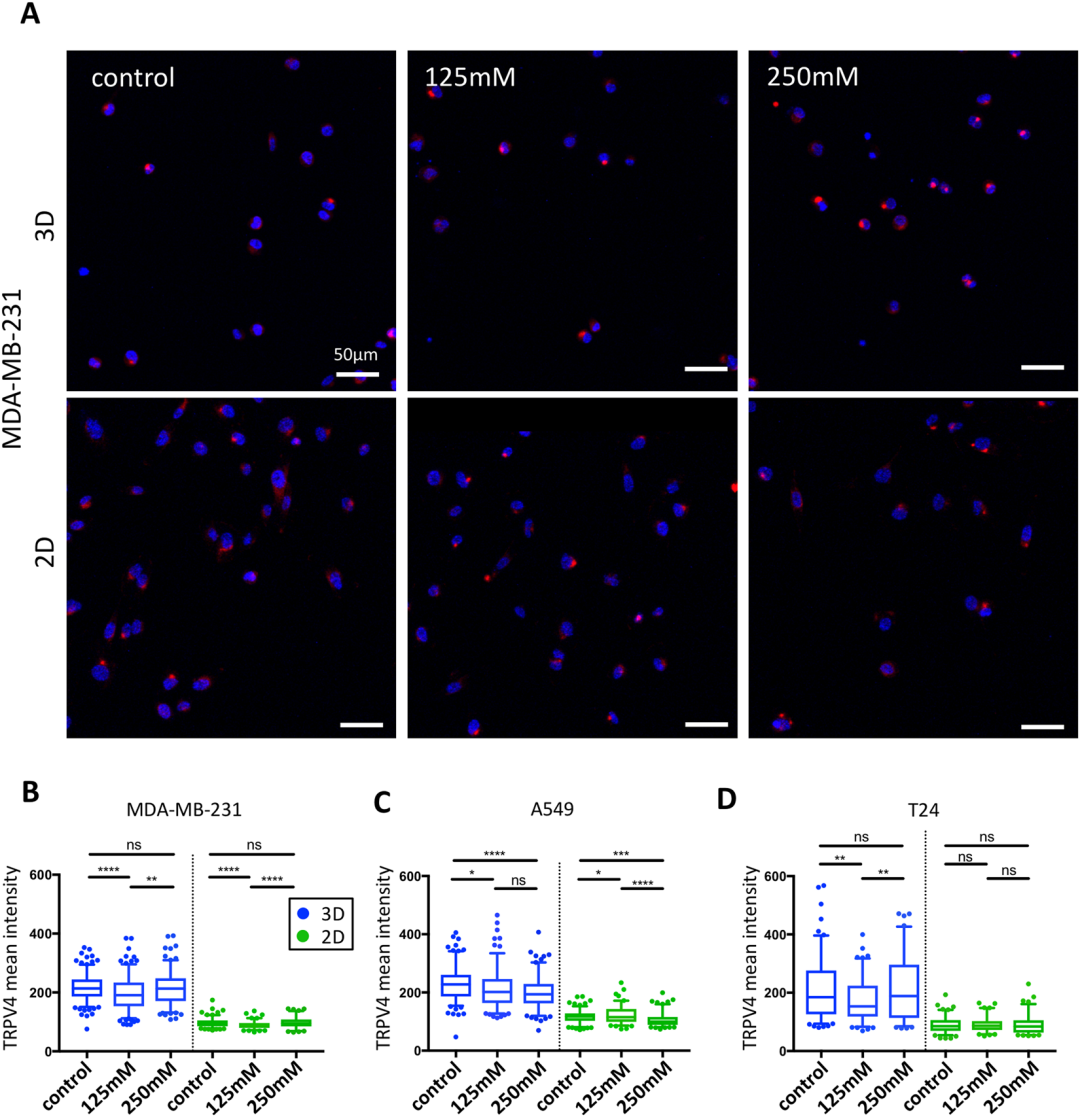
Changes in TRPV4 expression under hyper-osmotic stress in 3D and 2D conditions. (**A**) Immunofluorescence staining of TRPV4 in MDA-MB-231 cells under different hyper-osmotic conditions shows a spot-like staining corresponding to the accumulation of TRPV4 into an intra-cellular compartment. Cell nuclei are in blue and TRPV4 in red. (**B**–**D**) Averaged TRPV4 intensity per cell for MDA-MB-231 (**B**), A549 (**C**) and T24 cells (**D**) in either 3D (blue plots) or 2D (green plots) environments. The median (whisker boxes) and 5^th^ to 95^th^ percentile (lines) are shown for each condition. Legend box is shown once for all graphs.

Total TRPV4 expression presented minor variations under hyper-osmotic stress for all three cell lines in both 2D and 3D environment (Fig. 3A–D). Still, these changes observed were different for each cell line and did not show consistent trends. In contrast, a clear change in TRPV4 intra-cellular localization was observed in the 3D environment (Figs 3A, S3). Specifically, TRPV4 displayed a diffuse cytoplasmic localization in the majority of cells in the control condition (no osmotic stress applied). After hyper-osmotic induction, TRPV4 was seen in bright spots, characteristic of an intra-cellular aggregation (Figs 3A, S3 and S4). Similar observations were made for the three cell lines tested, but with higher clarity in MDA-MB-231. No clear differences in TRPV4 localization could be observed between the different osmotic conditions in 2D.

### Impact of hyper-osmotic stress on the cell dispersion from aggregates

**Figure 4:**
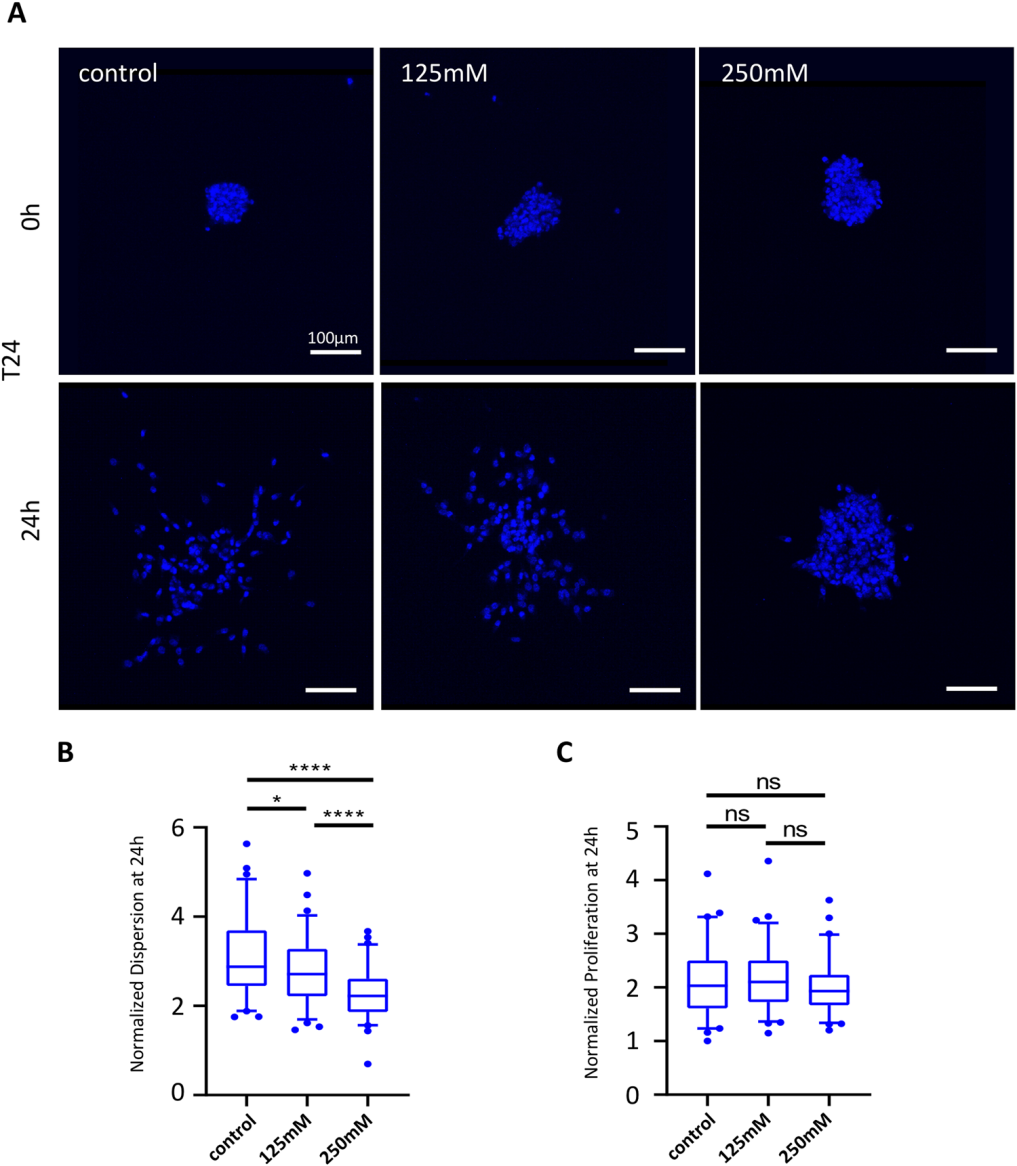
Effect of hyper-osmotic induction on the cell dispersion and proliferation of T24 aggregates. (**A**) Representative confocal images of T24 aggregates at 0 h and 24 h after exposure to different osmotic conditions. Cell nuclei are shown in blue. (**B**) Aggregate dispersion (normalized to the initial aggregate compactness at 0 h) and (**C**) proliferation rate of T24 aggregates after 24 h of exposure to different osmotic conditions. The median (whisker boxes) and 5^th^ to 95^th^ percentile (lines) are shown for each condition.

In order to better characterize the effect of hyper-osmotic stress on tumors, we applied the above-described range of hyper-osmotic stresses on human bladder carcinoma T24 aggregates and quantified their dispersion. We observed a significant dose-dependent decrease in cell dispersion for aggregates under heperosmotic stress compared with control aggregates (Fig. 4A,B). One could argue that such an observation reflects a change in cell proliferation, as previously observed in this study concerning the behavior of single cells in a 3D environment. However, we verified that there was no significant difference in cell proliferation between the aggregates treated with hyper-osmotic conditions and the control (Fig. 4C). Therefore, the change in aggregate dispersion that was observed during the 24 h are likely to be independent from the proliferation activities of the T24 aggregates.

## Discussion

The present study provides a comparative analysis of cell migration, proliferation and ion channel/transporter modifications in response to hyper-osmotic stress for three different human cancer cell lines cultured in both 3D and 2D conditions.

It has been extensively described that the first cellular response to hyper-osmotic stress in a 2D condition is volume decrease followed by the disruption of different cellular structures such as the actin cytoskeleton^39,40^. On a longer time-scale, NaCl and organic osmolytes enter the cell thus triggering an inward flow of water; this process is termed regulatory volume increase (RVI) and permits cell volume recovery^40,41^. Therefore, we expected that the RVI mechanism allows cells to adapt to the change of environment within the 24 h following exposure to osmotic stress and to recover their pre-stress volume. However, MDA-MB-231 and A549 cell line embedded in the collagen matrix (3D) presented a slight increase in cell volume after 24 h of moderate osmotic stress (Fig. S1A–C). Our observations are still in accordance with previous cell volume recovery data which report that osmotically-stressed cells can present an adapted volume that is greater than the initial pre-stress volume^41,42^. The smaller volume of T24 cells embedded in 3D matrix observed 24 h after exposure to stress could reflect a different RVI efficiency. No significant volume changes could be observed for cells in 2D, suggesting that the volume recovery mechanism of cells seeded on flat substrates is more efficient than the same mechanism happening in cells embedded in a 3D matrix.

Despite cancer cells capacities to cope with volume changes, we showed that hyper-osmotic stress indeed impacts cancer cell migration and proliferation (Fig. 1B–G) in 3D and 2D conditions and for the three metastatic cell lines tested, with cell proliferation slightly more affected in 2D than in 3D.

Such results are in accordance with previous studies describing how cells in 3D or 2D environments have different proliferation rates as a result of alternative pathway activation^43,44^. Such differences mainly originate because of distinctive microenvironment composition and are cell-line-specific^25^. Similarly, we observed that the three metastatic cell lines investigated in this study presented a generally slower migration speed in a 3D setting compared to a 2D environment, in all the osmotic conditions tested. Such difference might be explained by various factors, for example the resisting forces imposed by the surrounding 3D matrix to the cells which depend on pore size, matrix density, matrix alignment and degradation properties, or inherent differences in the way that factors such as matrix stiffness and adhesive ligand density and composition affect migration in the two cases^24,45,46^. However, different sensitivity can be appreciated, with MDA-MB-231 cells presenting a more significant decrease (Fig. 1B,E). Different cell susceptibility to hyper-osmotic stress have been previously described as being dependent on cancer cell aggressiveness. For example, La Porta *et al*. showed that metastatic cells subjected to 1 kPa osmotic pressure are able to migrate more and pass more easily through fixed size pores than primary tumor cells exposed to the same condition^12^. In our study, MDA-MB-231, A549 and T24 are all metastatic cell lines, and the differences observed may more likely originate from differences in their tumor progression mechanisms.

We then explored the impact of hyper-osmotic stress on the regulation of ion channels/transporters that are involved in tumor progression and cell invasion. A decrease in AQP5 expression has been previously described in the MCF-7 cell line subjected to hyper-osmotic stress^20^. Similarly, we observed a decrease in AQP5 expression in the MDA-MB-231 cell line (Fig. 2A,B). However, for A549 and T24 cell lines, changes in AQP5 expression were only detected in the 3D condition under severe induction of osmotic stress (Figs 2C,D and S2). This is in accordance with our previous migration and proliferation data, showing that MDA-MB-231 cells are more sensitive to hyper-osmotic stress than A549 and T24 cell lines. The change in TRPV4 expression was not as significant as AQP5 for the different conditions and cell lines and did not show a clear trend (Fig. 3). However, osmotic stress induced an increased translocation of TRPV4 from a diffuse cytoplasmic localization to a cytosolic compartment close to the nucleus (Figs 3A, S3 and S4). Change of localization of TRPV4 has also been previously observed in cardiac myocytes in response to hypotonic stimulation^47^. We could infer that the targeted organelle in our study could be the Golgi apparatus since TRPV4 has been previously observed to localize to this compartment in cultured human endothelial cells^48^ but this hypothesis would need further investigation. However, it is not clear why TRPV4 translocated under osmotic induction. The absence of modifications for AQP5 in A549 and T24 cells as well as TRPV4 in 2D environment could reflect differences in both osmoregulation and migration behavior when cells are studied on flat substrates or 3D matrix. In 3D, it is likely that transporters and aquaporins play a more prominent role for cell migration than in 2D. In agreement with this hypothesis, Stroka *et al*. showed that metastatic cells embedded in small 3D channels could solely rely on the activity of membrane transporters to trigger an osmotic gradient and ultimately cell displacement^23^. This migratory strategy, termed the osmotic engine model, is actin and myosin independent, and relies on fluxes of ions and water in/out the cell through the activity of passive ion channels, active ion pumps and aquaporins.

Finally, the response of aggregates embedded in 3D collagen gel to osmotic stress was also investigated. Research on cancer aggregates has given rise to new insights into the processes of cancer metastasis. These aggregates share common features with *in vivo* carcinomas and provide a physiologically improved platform for studying the chemo- and radio-resistance of tumors^49^. Previously, Montel *et al*. questioned the impact of mechanical forces on the growth of aggregates and described a drastic inhibition of cell proliferation^14^. However, their method did not involve hyper-osmotic shock but instead consisted of applying uniform mechanical compression on only the surface of the aggregates. In this study, we observed that aggregate dispersion but not proliferation is affected by hyper-osmotic stress, therefore demonstrating that aggregates react differently to hyper-osmotic stress than to mechanical compression.

To conclude, despite similar proliferation and migratory responses of single metastatic cell lines cultured in 2D and 3D to hyper-osmotic stress, noticeable differences were observed in the expression and the intra-cellular localisation of, respectively, AQP5 and TRPV4, both of which are involved in osmoregulation and cancer progression.

## Conclusion

Using an *in vitro* microfluidic device, the impact of hyper-osmotic environment on cancer cell was investigated, exploring how mechanical stress impacts cancer cell progression differently whether cells are cultured in 2D or 3D settings. Although some previous studies on osmotically- stressed cancer cells have been performed in 3D, those models lack the precision to fully recapitulate the cancer environment. Additionally, those analyses often focused on one cancer cell line and one level of osmotic stress - therefore possibly missing dose-dependence responses. Despite similar proliferation and migratory reactions of single metastatic cell lines cultured in 2D and 3D to hyper-osmotic stress, noticeable differences were observed in the expression and the intra-cellular localisation of, respectively, AQP5 and TRPV4, both of which involved in osmoregulation and cancer progression. Moreover, T24 aggregates do not display observable differences in their proliferation capacity - unlike single metastatic cells – but exhibit substantial differences in their ability to disperse. To conclude, this study unravels the variability in osmoregulatory mechanisms of different metastatic cell lines, not only based on their intrinsic properties, but also on their culture conditions in addition to being either cultured as single cells or aggregates. Therefore, this study highlights the importance of using a 3D assay to investigate certain physiological aspects of cancer progression. Moreover, a better understanding of the osmoregulation of cancer cells in different *in vitro* conditions might help to better target and hence interfere with the interactions and communication cues of cancer cells with their microenvironment.

## Materials and Methods

### Cell culture and media

Human mammary adenocarcinoma cells MDA-MB-23, human lung adenocarcinoma cells A549 and human urinary bladder carcinoma cells T24 (ATCC) were cultured in Dulbecco’s Modified Eagle Medium (DMEM; Invitrogen) supplemented with 10 % Fetal Bovine Serum (FBS; Invitrogen), 1 % L-glutamine and antibiotics. Cell cultures were kept in a humidified incubator maintained at 37 °C and 5 % CO2 and the medium was replaced daily. Hyper-osmotic stress was applied using mannitol (Sigma). A master solution of 1 M was prepared with complete media and solution of 125 mM (469 mOsm/kg H20) and 250 mM (608 mOsm/kg H20) were prepared for each experiment. The osmolarity of each solution was measured using freezing point depression with an Advanced Instruments Micro-Osmometer (Precision System, OSMETTE, Model 5004, Natick, MA, USA).

### Formation of cancer cell aggregates

T24 cells were used to form cell aggregates in a laser-patterned custom-made 60-mm polystyrene petri dish as previously described^49^. Prior to the addition of T24 cells, the microwell dishes were cleaned with 70 % ethanol and sterilized by UV-laser. In order to prevent cell attachment to the substrate, the microwell dishes were treated for 1 h with a 0.2 % pluronic solution in 1X PBS (Pluronic F108; Sigma-Aldrich, 542342). 5 × 10^5^ cells were added dropwise into the petri dish (Dow Corning, 430589) and maintained with 5 mL of DMEM medium at 37 °C and 5 % CO_2_ for 4 days. Cell aggregates were retrieved from the microwell dish and sieved through two cell strainers to yield aggregates with diameters in the range 40–70 µm. The aggregates were then enriched by centrifugation.

### Cell seeding into the microfluidic device

The commercially available microfluidic device DAX-1 (DAX-1, AIM BIOTECH) was used as previously described^28,50^. Briefly, the microfluidic layout consists of a central gel region (width 1.3 mm) surrounded by two media channels (width 0.5 mm). Rat tail collagen type I (BD Biosciences) solution was prepared at a concentration of 1.8 mg/ml for single cells and 2.5 mg/ml for T24 aggregates mixed with 10 x PBS and NaOH (0.5 N). Both single cells or T24 aggregates were added to the mixture at a 1:10 ratio (5 × 10^5^ cells/ml). 10 μl collagen mix containing single cells or aggregates were then injected into the gel channel and incubated at 37 °C to allow polymerization by thermal cross-linking. In order to avoid cells from concentrating at the bottom surface of the channel, the device was turned over manually three times every 5 min at room temperature and then left unturned for 15 min in 37 °C, 5 % CO_2_. The media channels were then filled with DMEM medium. In order to label the nucleus to track cell migration, NucBlue™ Live Cell Stain (Life Technologies, Grand Island, NY) was added to the media for live staining following the manufacturer’s protocol. The device was incubated at 37 °C for 3 h prior to induction. Hyper-osmotic stress was applied by replacing DMEM media with the respective mannitol concentrations. The control channel was replaced using normal DMEM media.

### Immunostaining

Cells in the device were fixed by replacing the medium from the microfluidic device with 1X PBS, then with 4 % formaldehyde (Sigma-Aldrich) for 15 min at room temperature. The device was washed twice in 1X PBS and stored at 4 °C. Cells were permeabilized with 0.5 % Triton X-100 (Sigma-Aldrich) for 15 min. For proliferation assays, fixed and permeabilized cells were incubated with 10 µg/ml Ki67-FITC (clone SolA15; eBioscience) and NucBlue™ Live Cell Stain in 0.5 % BSA + PBS for 3 h at RT, then washed several times with 1 x PBS prior to imaging. F-actin was stained using ActinGreen 488 ReadyProbes Reagent (Life Technologies) and NucBlue™ Live Cell Stain few hours before imaging. For immunostaining, the devices were washed twice in 1X PBS before incubating the cells for 3 h with a 3 % BSA blocking solution. After blocking, samples were incubated overnight at 4 °C with either rabbit polyclonal antibody either against AQP5 at 1:50 dilution (Thermofisher) or TRPV4 at 1:100 dilution (Thermofisher) in 0.5 % BSA. The secondary antibody used was Alexa Fluor 647 anti-rabbit (1:200, Life Technologies) containing 0.5 % BSA and NucBlue™ Live Cell Stain. After 1 h incubation at room temperature, the devices were washed with 1X PBS and imaged.

### Image acquisition and processing

All images were captured using a confocal microscope (Olympus IX81). For time-lapse migration assay acquisition of cells in both 3D and 2D conditions, an environmental chamber attached to the microscope allowed for cells to be maintained at 37 °C and 5 % CO_2_. The cells were induced for 24 h with specific osmotic conditions prior to imaging. Cancer cell migration was acquired by capturing image stacks (5 μm slice thickness) every 20 min with 512 × 512 pixel resolution for up to 12 h using a 20 X objective lens. The mean migration speed was measured using Imaris software (Bitplane Scientific Software) by calculating the recorded trajectory of individual nuclei as previously described^51^. Briefly, the nucleus of each cancer cell was identified and processed using the spot tracker function. The mean migration speeds were then averaged for all cancer cells.

T24 aggregates were acquired with a 20 X objective lens (5 μm slice thickness) at 0 h and 24 h after hyper-osmotic induction. Three experiments were independently conducted and for each of them, aggregates were acquired from three devices per condition. Aggregate dispersion (normalized to the initial aggregate compactness at 0 h) was quantified using Imaris software as previously described^50^.

For proliferation assays, z-stacks were acquired with a 10 X objective lens. Target cell proliferation was quantified by measuring the proportion of FITC cells among the nuclear-labelled cells (NucBlue). Immunostained cancer cells were acquired with a 20 X or 60 X objective (3 μm-thick slice) with 640 × 640 pixels resolution. Three fields of view were acquired per condition and averaged for each experiment. Quantification was performed by measuring the total mean intensity per cell without performing image corrections since 3D and 2D image background intensity showed no significant difference (P > 0.05; unpaired Student’s t-test; data not shown). All the experiments were performed in triplicate.

### Statistical analysis

For Fig. 1E–G, data were presented as the mean ± SEM. For all other figures, values were presented as the median (5^th^ to 95^th^ percentile). At least three independent devices were used for each condition. Quantitative data were compared by performing unpaired Student’s *t*-test. ^∗^*P* < 0.05 was considered as significant. All statistical analyses were done using GraphPad Prism (GraphPad Software, Inc., San Diego, CA).

## Supplementary information

SUPPLEMENTARY INFO Quantitative screening of the effects of hyper-osmotic stress on cancer cells cultured in 2- or 3-dimensional settings
