# Supplementary material for: Quantitative screening of the effects of hyper-osmotic stress on cancer cells cultured in 2- or 3-dimensional settings
Source: Sci Rep. 2019 Sep 24;9:13782. doi: 10.1038/s41598-019-50198-w (PMC6760113; doi:10.1038/s41598-019-50198-w)
Supplement: Supplementary file 1 — SUPPLEMENTARY INFO Quantitative screening of the effects of hyper-osmotic stress on cancer cells cultured in 2- or 3-dimensional settings [file 41598_2019_50198_MOESM1_ESM.pdf]

Quantitative screening of the effects of hyper-osmotic stress on cancer cells cultured in 2- or 3-dimensional settings

Agnes Miermont[1], Sharon Wei Ling Lee[2,3,4], Giulia Adriani[4,5] & Roger D. Kamm[6]

[1] Stem Genomics, IRMB, Univ Montpellier, INSERM, CHU Montpellier, Montpellier, France.

[2] BioSystems and Micromechanics, IRG, Singapore-MIT Alliance for Research and Technology, Singapore

[3] Department of Microbiology and Immunology, Yong Loo Lin School of Medicine, National University of Singapore, Singapore

[4] Singapore Immunology Network (SIgN), Biomedical Sciences Institute, Agency for Science, Technology and Research (A*STAR), Singapore

[5] Department of Biomedical Engineering, Faculty of Engineering, National University of Singapore, Singapore, Singapore.

[6] Department of Biological Engineering, Massachusetts Institute of Technology, Cambridge, MA, United States

Agnes Miermont and Sharon Wei Ling Lee contributed equally.

Correspondence and requests for materials should be addressed to R.D.K. (email: rdkamm@mit.edu)

Supplementary Figures:

Supplementary Figure 1:

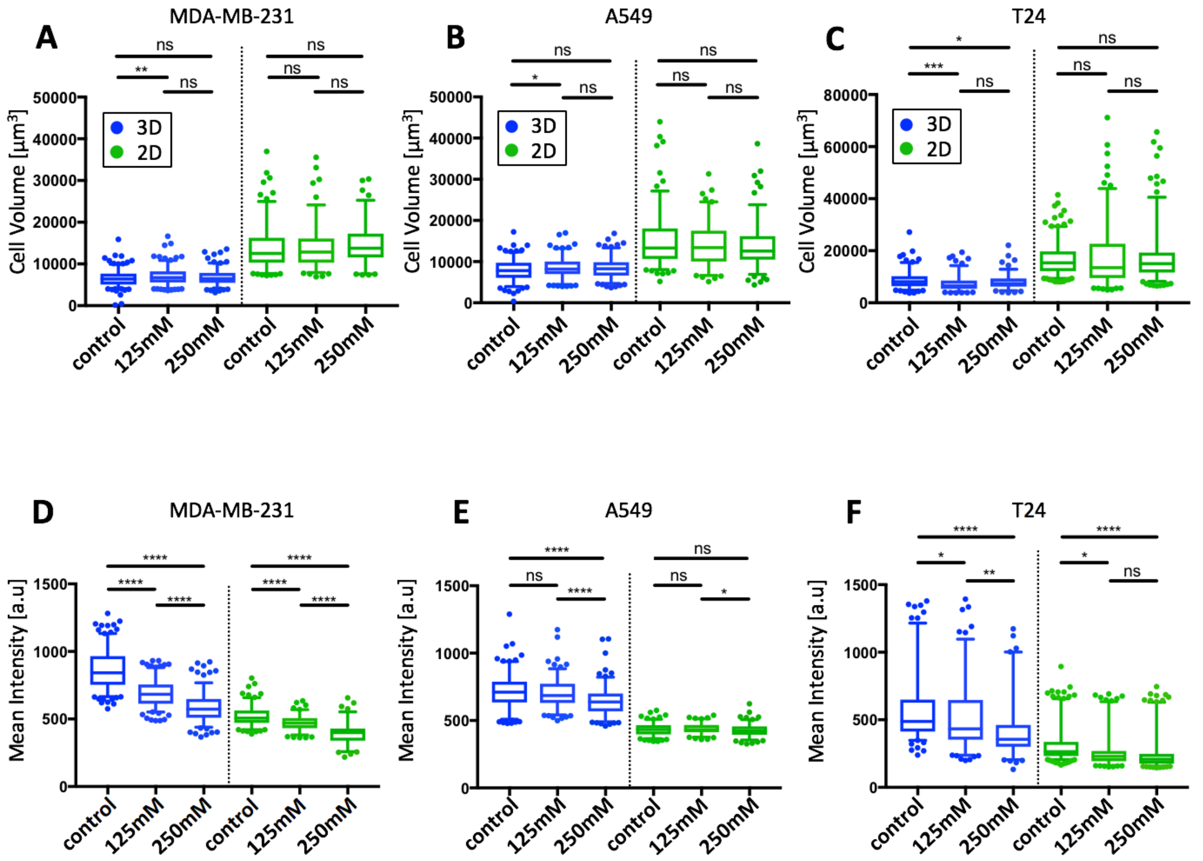

Figure S1: Impact of hyper-osmotic stress on cell volume and actin expression. (A-C) Cell volume measurement for MDA-MB-231 (A), A549 (B) and T24 (C) cells after 24 h of osmotic induction, in either 3D (blue plots) or 2D (green plots) conditions. D-F) Actin intensity measurement for MDA-MB-231 (D), A549 (E) and T24 (F) cells after 24 h of osmotic induction in either 3D (blue plots) or 2D (green plots) conditions. The median (whisker boxes) and 5th to 95th percentile (lines) are shown for each condition. Legend box is shown once for each cell line.

Supplementary Figure 2:

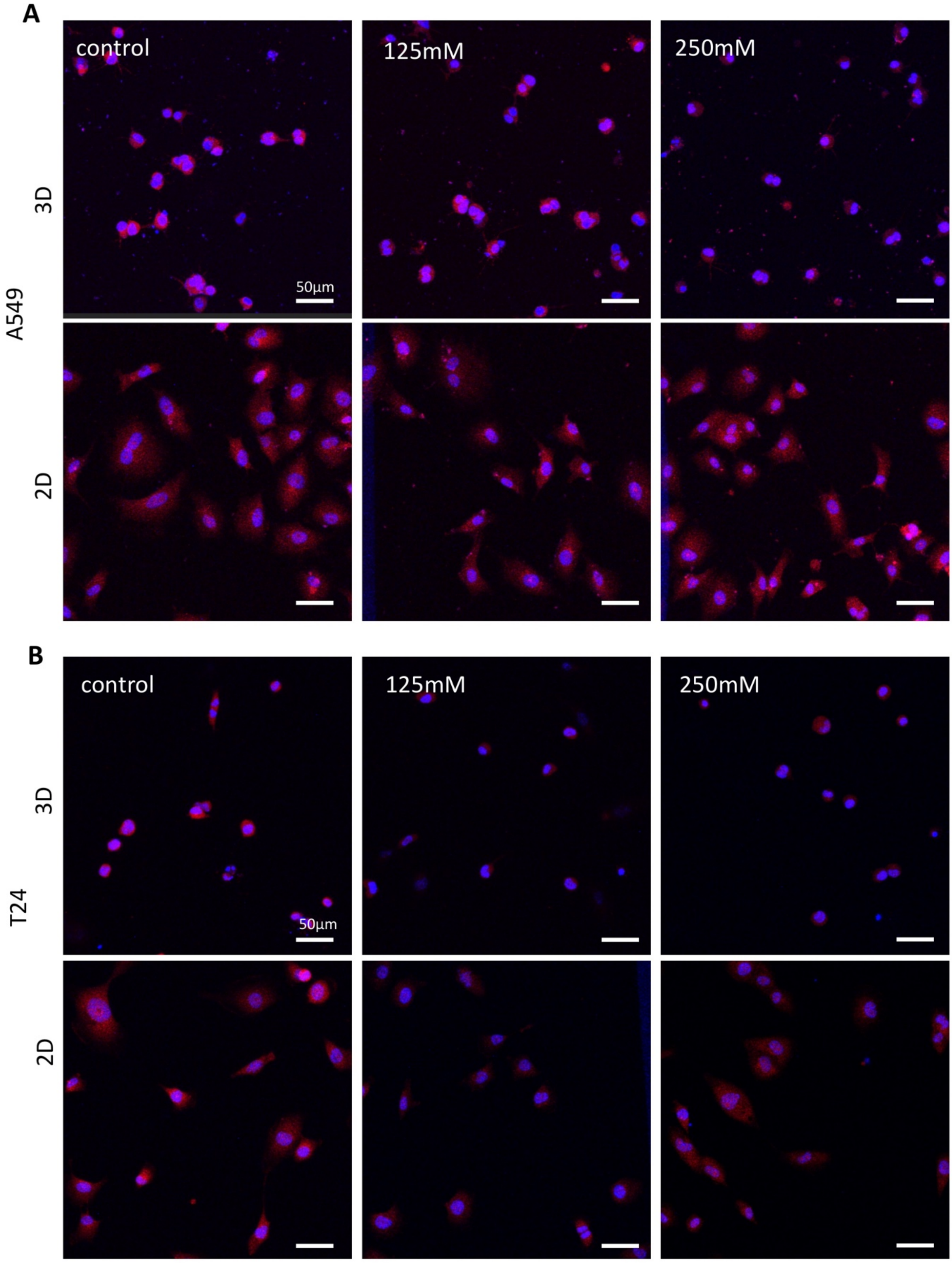

Figure S2: Immunofluorescence staining of AQP5 in A549 cells (A) and T24 cells (B) under different osmotic conditions in either 3D (top) or 2D (bottom) environment. Cell nuclei are shown in blue and AQP5 in red.

Supplementary Figure 3:

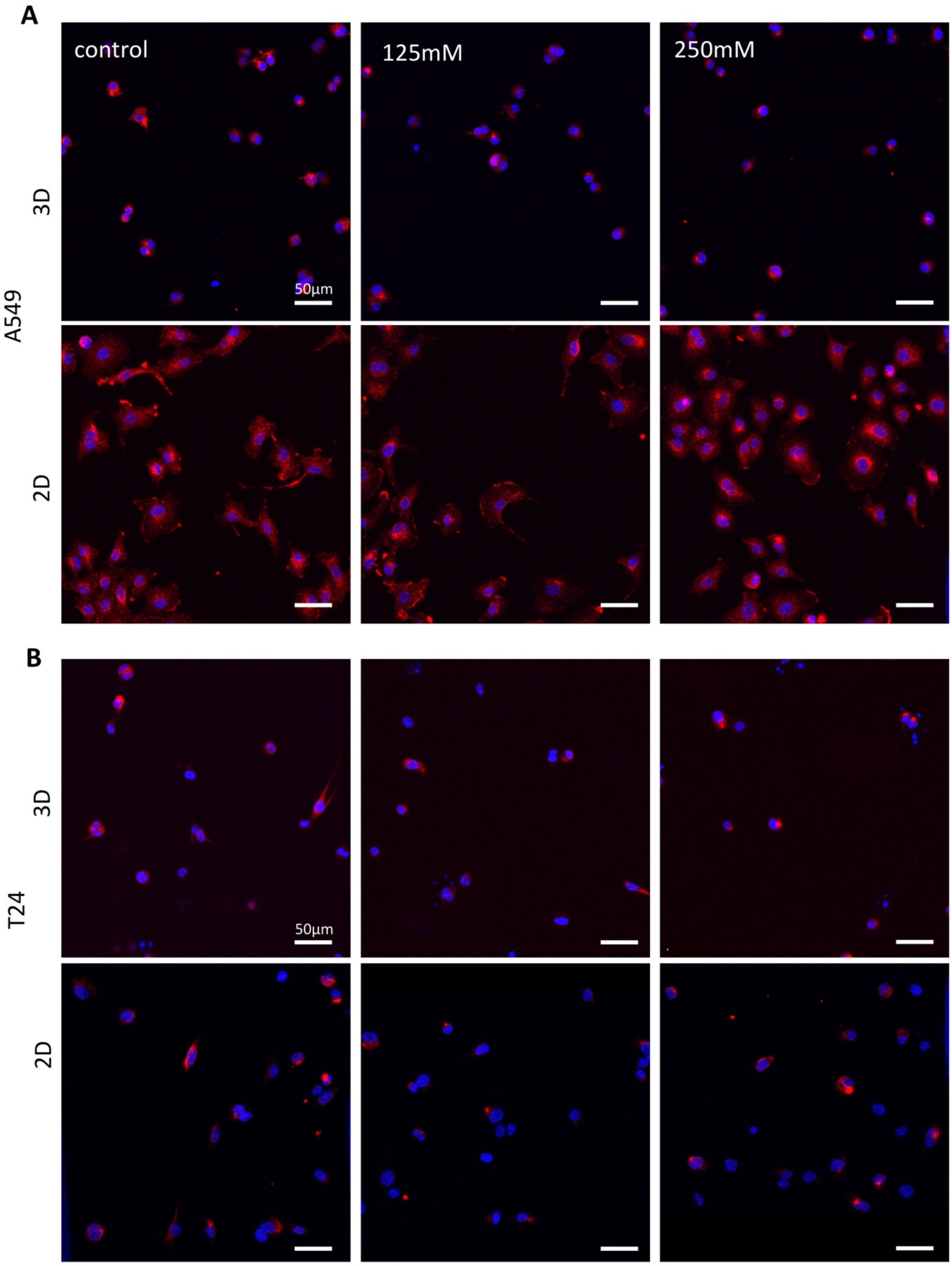

Figure S3: Immunofluorescence staining of TRPV4 in A549 cells (A) and T24 cells (B) under different osmotic conditions in either 3D (top) or 2D (bottom) environments. Cell nuclei are shown in blue and TRPV4 in red.

Supplementary Figure 4:

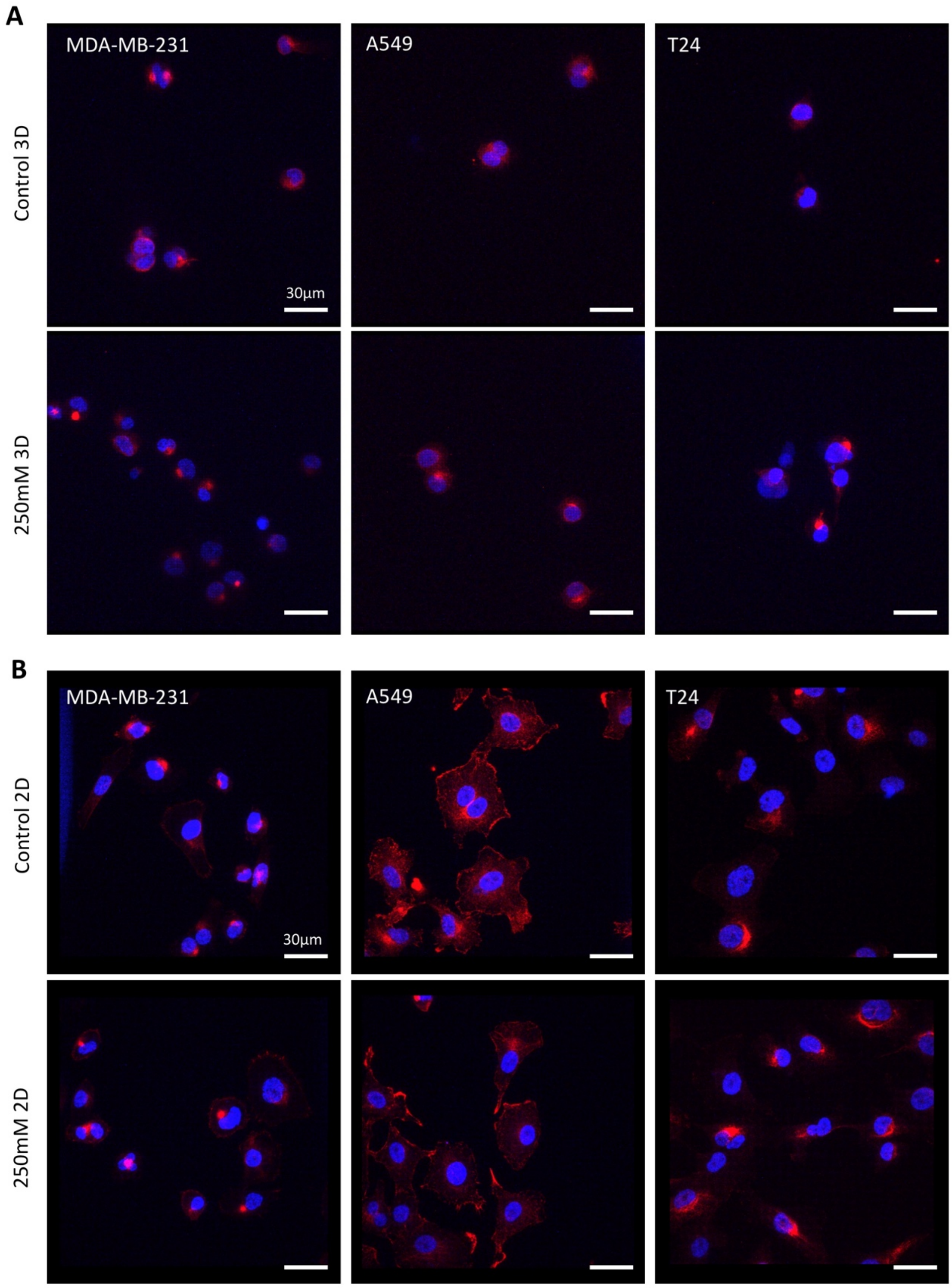

Figure S4: Immunofluorescence staining of TRPV4 using 60X magnification for the three metastatic cell lines for better visualization and comparison of control versus severe osmotic condition (250 mM mannitol – 608 mOsm/kg H20) in either 3D (A) or 2D (B) environments. Cell nuclei are shown in blue and TRPV4 in red.
